# Special Issue Editorial: Emerging Therapies in Renal Cell Carcinoma: The Road to a Cure?

**DOI:** 10.3390/cancers15215262

**Published:** 2023-11-02

**Authors:** Matthew T. Campbell, Tian Zhang

**Affiliations:** 1 Department of Genitourinary Medical Oncology, University of Texas M.D. Anderson Cancer Center, Houston, TX 77030, USA; 2 Department of Internal Medicine—Hematology/Medical Oncology, University of Texas Southwestern, Dallas, TX 75235, USA; tian.zhang@utsouthwestern.edu

## 1. Introduction

In the past two decades, therapy development in the treatment of renal cell carcinoma has exploded. In our Special Issue on cancers, “Therapy Development in Renal Cell Carcinoma”, the decision to use therapy as opposed to treatment was intentional. Current multidisciplinary therapies include surgical intervention, radiation therapy, interventional ablative techniques, and systemic therapy utilizing immunotherapy and targeted therapy. In many patients who develop metastatic disease, a combination of these strategies with experienced providers that recognize the opportune times to employ interventions, change therapy, and optimize patient partnership can lead to better outcomes.

Starting with surgery, the last twenty years has seen improvements in patient selection, anesthesia, post-operative recovery pathways, and operative techniques, including the employment of robotic surgery. Evolution in understanding the optimal candidates for partial nephrectomy versus radical nephrectomy, the management of multiple tumors, bilateral tumors, tumor thrombus, impacts on renal function and co-morbidities, and the deferral of cytoreductive nephrectomy in most patients has been championed by urologic oncologists. In patients with metastatic clear cell renal cell carcinoma, the role of cytoreductive nephrectomy remains under intense debate. While the CARMENA study helped to prospectively define that patients with poor-risk disease are not appropriate for upfront cytoreductive nephrectomy, it did not address the role of delayed cytoreductive nephrectomy in responding patients [1]. An ongoing study called PROBE (NCT04510597), randomizing patients on immunotherapy to nephrectomy versus continuing immunotherapy, will help to address the role of early nephrectomy. However, this study will not address patients who later progress in their primary tumor, those who become symptomatic with bleeding or pain, or the optimal timing of nephrectomy. Improved imaging modalities are clearly needed to help make more informed decisions. Potential improvements in detecting circulating tumor DNA and its evolution may also play a role in these decisions. Multidisciplinary conferences remain crucial to help guide current patient selection.

In addition to addressing primary renal tumors, surgical decisions about metastatic disease sites remain critically important. Patients with oligometastatic disease, a somewhat shifting target in definition, have long been known to potentially benefit from metastasectomy. While these procedures are often not curative, they hold the potential to delay the need for systemic treatment or provide a systemic treatment break. Importantly, these procedures can also potentially prevent catastrophic consequences for patients, including large symptomatic brain metastasis and skeletal-related events for those with bone metastases, particularly involving long bones or patients experiencing vertebral column instability. 

Moving toward radiation therapy, renal cell carcinoma has gone from being labeled as a radiation-insensitive tumor to a cancer that can be addressed with hypo-fractionated and stereotactic radiation delivery. As addressed in this issue by Christensen et al., radiation therapy can be used to treat primary tumors, tumor thrombus, and can be considered for appropriately selected metastatic sites in oligometastatic, symptomatic, or high-risk sites including brain metastases and bone metastases [2]. It can also be used to potentially consolidate a great treatment response and is being formally tested to delay the time until systemic therapy for patients with oligometastatic RCC (NCT05863351) and EXTEND (NCT03696277) the response in patients who begin to progress upon systemic therapy in limited sites. With the completion of these trials, oligometastatic and oligo progressing metastatic RCC can become a multidisciplinary disease state.

Interventional radiology techniques have also evolved with aids to diagnoses, the use of ablative techniques, and the delivery of drugs, immune stimulants, and radiopharmaceuticals. Historically, in patients with primary renal tumors, the safety of biopsy was questioned, given concerns about bleeding or tumor seeding. In a review by Volpe A et al., the risk of major complications was less than 1%, allowing for histological confirmation to help guide therapy decisions, including active surveillance [3]. In this issue, Abdelsalam M et al. reported 20-year outcomes for the radiofrequency ablation of T1A renal tumors at the MD Anderson Cancer Center [4]. The authors reported (at a median follow-up of 3.7 years) a 96.5% local control rate, 88.6% disease-free survival rate, and 100% overall survival rate. Ablative techniques are also used in the treatment of oligometastatic and symptomatic metastatic lesions, or to help consolidate tumor control in high-risk sites, including liver and bone. Additional interventional radiology techniques, including the management of symptomatic or high-risk bone tumors, including kyphoplasty with cement, can be crucial in addressing vertebral body metastases or diminishing the risk of vertebral body fractures after the use of stereotactic radiation on the spine. The role of the delivery of radiopharmaceuticals in the treatment of metastatic renal cell carcinoma has not been prospectively evaluated, but retrospective reports of the utility of Yttrium-90 microspheres (Y-90) in patients with a liver-dominant presentation have been reported [5]. Continued engagement with our interventional radiology colleagues remains crucial in continuing to advance novel drug delivery techniques.

Moving toward drug development for metastatic clear cell renal cell carcinoma (mccRCC), a profound evolution in outcomes is evident when comparing the initial publication by Motzer RJ et al. in 1999 (when the MSKCC criteria was established) to the 67-month follow up from the CheckMate 214 study [6,7]. In 1999, the median survival in a study of interferon alpha was only 10 months, compared to 55 months for the intention to treat the population from CheckMate 214. This drastic improvement in overall survival is the result of two decades of research on the development of targeted therapy focused on vascular endothelial growth factor receptor tyrosine kinase inhibitors (TKI), mammalian target of rapamycin inhibitors (mTOR), and an immune checkpoint blockade focused on cytotoxic T lymphocyte antigen-4 (CTLA-4) and programmed death receptor 1 (PD-1) antibodies. A major salute must be given to all those in medical oncology focused on renal cell carcinoma in the cytokine era (prior to 2005), as trial after trial testing chemotherapy, including high-dose chemotherapy with autologous stem cell rescue, radiation, and vaccines, were met with failure. Dr Rosenberg and his team worked tirelessly at the National Institute of Health and through the Cytokine Working Group to establish high-dose interleukin 2 as a strategy capable of curing 5–7% of patients with mccRCC, but with high associated toxicities, requiring skillful monitoring often in intensive care with a risk of mortality [8]. The development of TKI and mTOR inhibitors began a march of FDA-approved therapies that consistently improved progression-free survival, but through to 2015, could not improve overall survival outside of temsirolimus in poor-risk metastatic disease [9]. 

However, it was clear that patients were living longer with the important collaboration with the International Metastatic Renal Cell Carcinoma Database Consortium (IMDC). In 2009, the initial publication of the IMDC criteria for prognostication in the “targeted therapy era” found that the median overall survival had improved to 22 months [10]. Clear improvements in outcomes were seen in patients with favorable and intermediate risk, with a relative lack of improvement in patients with poor-risk disease. Drugs approved prior to 2016 include sorafenib, sunitinib, axitinib, pazopanib, temsirolimus, and everolimus. By 2015, sunitinib and pazopanib were considered to be standard front-line agents, with temsirolimus being the only agent tested in poor-risk disease. By 2015, sorafenib, axitinib, and everolimus were used in second- or later-line settings, with many questions about the optimal sequence. 

In 2016, three studies introduced agents that have ushered in a new era of treatment and led to FDA approvals in the post-TKI setting. Nivolumab (nivo), the first PD-1 agent approved, cabozantinib (cabo), and lenvatinib (len), in combination with everolimus, all established active regimens, with the first two showing overall survival benefits as compared to treatment with everolimus [11]. Beginning in 2018 through to 2021, five frontline combination regimens received FDA approval, with nivo plus ipilimumab (ipi), pembrolizumab (pembro) plus axitinib, avelumab plus axitinib, nivo plus cabo, and pembro plus len [12,13]. Except for avelumab plus axitinib, which met its primary endpoint of improvement in progression-free survival, but not overall survival, all four other regimens improved overall survival when compared to front-line treatment with sunitinib. Based on the design of the study with nivo plus ipi, the primary endpoint focused on patients with intermediate- and poor-risk disease, as defined by the IMDC criteria, while the other studies included all subgroups in their primary endpoint, thus leading to a differential in category recommendation by the NCCN guidelines.

In this issue, Zarrabi et al. walked through the complicated decision tree required to select the optimal front-line therapy in mccRCC [14]. Since the approval of nivo plus ipi, a clearer picture of the ideal candidates seems to be emerging. Ipi is most active in the front-line setting, with several later prospective studies finding a low rate of response and no complete responses. Patients with sarcomatoid de-differentiation are known to be TKI-resistant and have high response rates and complete response rates with nivo plus ipi. The clear allure and benefit of nivo plus ipi is an opportunity for durable remission with treatment-free survival. While additional follow-ups will be of major importance, a cure fraction in the neighborhood of 25% appears to be materializing from the CheckMate 214 study [7]. The downside of nivo plus ipi is a high rate of immune-related adverse events and the relatively high rate of primary progressive disease. In patients with high-volume disease in high-risk locations, including the liver, bone, brain, pleural effusions, and ascites, who have one attempt to obtain disease control, the use of nivo plus ipi is not ideal. The use of TKI+PD-1 has also revolutionized the front-line space. With pembro plus axitinib, pembro plus len, and nivo plus cabo, the initial response rate across regimens is well above 50%, with low primary progression rates close to 10%. In patients who have a high tumor burden and one therapeutic attempt for response, these regimens offer incredible disease control rates. What remains less clear is the optimal duration of the PD-1 agent and if a subset of these patients without substantial toxicity can discontinue therapy and experience treatment-free survival intervals without toxicity/side effects. Durable responses and cures remain goals of treatment, and ongoing trials such as PDIGREE (NCT03793166) and LITESPARK 012 (NCT04736706) are testing optimal sequencing approaches and intensifying treatment with triplet immunotherapy-based combinations, respectively. 

## 2. Adjuvant Therapy

In this issue, Berg et al. summarized the current landscape of adjuvant therapy in renal cell carcinoma. As in other solid tumors, the primary approach to localized RCC is surgical, with systemic therapies being tested in the adjuvant setting to improve time until disease recurrence (disease-recurrence-free survival rates). In the TKI era, several TKIs were tested in the post-operative adjuvant setting, and only sunitinib in the S-TRAC trial improved disease-free survival. One of these trials, ASSURE, along with other retrospective series, led to the creation of important disease-free survival nomograms/calculators considering the clinical and pathologic features of patients and their tumors at the time of nephrectomy. These continue to be used to aid in decision making in the adjuvant setting. 

In 2021, the adjuvant trial KEYNOTE 564 reported an improvement in the primary endpoint of disease-free survival when comparing the PD-1 inhibitor pembrolizumab to a placebo in the adjuvant setting [15]. Based on these data, pembrolizumab was approved for the adjuvant treatment of renal cell carcinoma at an intermediate or high risk of recurrence. However, overall survival (a secondary endpoint) has not matured enough to have a definitive impact for pembrolizumab. Along with three subsequent trials which were negative in terms of improving disease-free survival (Checkmate 914 combination ipi/nivo versus placebo, IMmotion 010 atezolizumab versus placebo, and PROSPER-RCC perioperative nivolumab before surgery versus nephrectomy), the lack of OS data from Keynote 564 has created the current controversy surrounding the absolute benefit of pembrolizumab [16,17,18]. Further, the optimal patient to select for treatment (high-risk, node-positive, or metastatic s/p metastasectomy) is still under intense debate. Ultimately, patient preferences and perspectives on the risk/benefit calculation should be carefully considered when deciding on the role of adjuvant pembrolizumab. 

## 3. Ongoing Therapy Development in mccRCC

Many systemic therapies are under clinical development and exploration for mccRCC outlined in this issue by Chen et al. [19]. These include the frontrunner HIF2a inhibitor belzutifan, along with multiple other HIF2a inhibitors under early-phase clinical trials. Belzutifan has been established as a therapeutic treatment which has changed the outlook for patients with VHL syndrome, substantially decreasing the numbers of surgeries, improving time until disease progression, and improving the objective responses for the population with hereditary VHL syndrome. Belzutifan is currently in late-phase clinical trials for refractory mccRCC, as well as in combination with pembro/len for front-line mccRCC, and finally in combination with pembrolizumab in adjuvant RCC.

Ultimately, to improve the treatment outcomes in immunotherapy-resistant mccRCC, mechanisms of resistance must be further understood. These mechanisms may include further tumor escape from the adaptive and innate immune system, subsequent mutations that affect targets in the angiogenic, or other mechanisms important in tumor metastasis. Cellular therapies tailored to the ccRCC tumor microenvironment, as well as novel immunotherapy targets, are in early-phase clinical development for refractory ccRCC. 

## 4. Non-Clear Cell with Focus on Papillary Carcinoma

The many distinct subtypes of non-clear cell RCC have been studied in aggregate in early trials of mTOR versus anti-VEGF TKIs, extrapolating from ccRCC knowledge. Given some of the challenges in accruing studies addressing non-clear cell RCC, many of the initial efforts enrolled multiple subtypes, including papillary, chromophobe, unclassified, and translocation. Two early phase II studies on everolimus versus sunitinib (ESPN and ASPEN trials) both supported the use of sunitinib [20,21]. While not powered to look at any of the subgroups individually, both ESPN and ASPEN hinted that chromophobe may be a subtype best treated with an mTOR inhibitor.

In this issue, Chawla et al. outlined important subsequent developments in papillary histology [22]. The PAPMET trial enrolled specifically metastatic papillary RCC and showed a benefit of cabozantinib over sunitinib in improving progression-free survival [23]. Cabozantinib is therefore the current standard of care for patients with metastatic papillary RCC, with the ongoing PAPMET2 trial enrolling and randomizing patients to cabozantinib with or without atezolizumab (NCT05411081). 

In the interim, the CONTACT-03 study had a small population with non-clear cell RCC which had prior immunotherapy exposure, and, in the intention to treat the population, did not show an improvement in cabozantinib-atezolizumab versus cabozantinib monotherapy. Several phase II single-arm studies have been completed, exploring len plus everolimus, nivo plus cabo, and pembro plus len. For len plus everolimus, the response rate was 28%, and an intriguing number of responses were seen in patients with chromophobe histology. Cabo plus nivo found activity with a response rate of 47% for patients in the cohort with papillary, unclassified, and translocation renal cell carcinoma, but no evidence of activity in the initial seven patients with chromophobe [24]. Pembro plus len had objective response rates of 55%, with evidence of the highest responses in papillary, unclassified, and translocation, but also a 28% response rate in the limited number of chromophobe patients under study [25]. 

As better biology is understood about each non-clear cell RCC subtype, further trials are currently underway to study each subtype. Ultimately, the biologies of papillary (FH loss or MET amplified) versus chromophobe versus medullary renal cancers are distinct and warrant further dedicated trials to improve the outcomes in each subtype.

## 5. Future Directions

The landscape for ccRCC has drastically changed, and patients with kidney cancer are living longer—hopefully with tolerable/manageable side effects. Patient advocates and patients themselves have declared the goals for ccRCC treatment to be cures and durable long-term survival without systemic side effects—while aspirational, this goal seems more attainable today than just 20 years ago. Imagine how far we as a field would proceed if our treatment advances improved the survival outcomes by five-fold in the next 20 years, as they have in the past 20 years. We have much more to learn about the resistance mechanisms, early disease detection, and optimal patient selection for sequential therapies, including the optimal sequencing of radiation and surgical approaches. As novel scientific discoveries are made in kidney cancer, we hold true to our patients’ hopes of cures and durable long-term survival, and imagine a day when our mutual hopes are fulfilled.
